# Early impairment of food intake in patients newly diagnosed with cancer

**DOI:** 10.3389/fnut.2022.997813

**Published:** 2023-01-05

**Authors:** Alessio Molfino, Sara Emerenziani, Giuseppe Tonini, Daniele Santini, Antonietta Gigante, Michele Pier Luca Guarino, Chiara Nuglio, Giovanni Imbimbo, Annalisa La Cesa, Michele Cicala, Maurizio Muscaritoli

**Affiliations:** ^1^Department of Translational and Precision Medicine, Sapienza University of Rome, Rome, Italy; ^2^Gastroenterology Unit, Campus Bio-Medico University, Rome, Italy; ^3^Oncology Unit, Campus Bio-Medico University, Rome, Italy

**Keywords:** cancer, food intake, early assessment, weight loss, cachexia, anorexia, hypophagia

## Abstract

**Background:**

Patients with gastrointestinal or lung cancer often suffer from a loss of appetite (anorexia), resulting in reduced food intake (hypophagia) and body weight loss. This study evaluated the prevalence of anorexia, hypophagia, pre-cachexia and cachexia in patients with cancer at time of diagnosis.

**Patients and methods:**

Patients with newly diagnosed gastrointestinal or lung cancers were included. Body mass index (BMI) and weight loss over the prior 6 months were recorded. Patients were assessed for (pre-)cachexia and for anorexia using the Functional Assessment of Anorexia/Cachexia Therapy (FAACT) and a specific anorexia questionnaire (AQ). Energy and protein intake were calculated through food diaries. Patients were considered hypophagic if intake was ≤70% of guideline-recommended levels.

**Results:**

Overall, 102 patients [53 male; median age: 67 (range, 21–88) years] were enrolled. Mean BMI (± standard deviation) was 23.1 ± 3.4 kg/m^2^; average percentage of weight loss was 10.1 ± 7.8%. At diagnosis, 68% (69/102) of patients had cachexia, and 11% (11/102) pre-cachexia. Prevalence of anorexia was 57% (58/102) and 75% (76/102) according to FAACT and AQ, respectively. Forty-eight percent (49/102) of patients had hypophagia. Patients with anorexia had lower daily energy (*p* = 0.002) and protein intake (*p* = 0.0257), and greater percentage of weight loss (*p* = 0.0005). In patients with hypophagia, negative correlations were observed between percentage of weight loss and total daily calorie (r = –0.40; *p* = 0.01) and protein intake (r = –0.340; *p* = 0.018).

**Conclusion:**

Anorexia, inadequate nutritional intake and cachexia are highly prevalent in patients with gastrointestinal or lung cancer at diagnosis. Negative protein and energy balance may play an important role in the pathogenesis of cachexia. Early multimodal strategies to improve food intake are urgently needed.

## Introduction

Cachexia is a main cause of morbidity and mortality in chronic conditions such as autoimmune disorders and cancer, particularly in late-stage disease (1). Cancer-associated cachexia is a multifactorial disorder characterized by body weight loss, including skeletal muscle and fat mass, anorexia, and metabolic and endocrine alterations, which cannot be fully reversed by nutritional support alone (2–4). Reduced food intake, a negative energy balance, and chronic inflammation are thought to play crucial roles in the pathogenesis of weight loss and cancer cachexia (3, 5).

The etiology of reduced food intake (i.e., hypophagia) associated with cancer is diverse. Tumor burden or chemotherapy may result in nausea, vomiting, or nutrient malabsorption (1, 6). Decreased upper gastrointestinal motility can also cause nausea and provide a sense of early satiety (7). Other potential causes include dysphagia, stomatitis, bowel obstructions, dyspnea, poor dietary habits, and hormonal changes (4, 6, 7), as well as pain, anxiety, and depression (6). Patients with cancer often also complain of loss of desire to eat, with anorexia further contributing to malnutrition and the onset of cachexia (8).

Cachexia has distinct tumor-driven components. Tumors undergo high rates of glycolysis and lactate production leading to high energy demands, and more-aggressive and advanced stages of cancer are associated with increased energy expenditure (9). Additional metabolic changes may be caused by the activation of the immune system, with chronic inflammation being linked to hypermetabolism (9). Tumor cells secrete pro-inflammatory cytokines that activate the immune system to induce a systemic inflammatory response. Catabolic pro-inflammatory factors acting in skeletal muscle, adipose tissue, and in the central nervous system (CNS) lead to an increase in energy expenditure (3, 8, 9). Of particular importance is the effect of chronic inflammation in the CNS, which can lead to anorexia, weight loss, skeletal muscle atrophy, and lipolysis (3). Based on this view, the action of pro-inflammatory molecules, particularly interleukin (IL)-6, in the hypothalamus may lead to an imbalance between appetite stimulants and suppressants, in turn resulting in anorexia and reduced food intake. In addition, IL-1β and tumor necrosis factor activity in the hypothalamus can trigger production of glucocorticoids by the adrenal gland, leading to skeletal muscle catabolism and rapid induction of atrophy (3).

The combination of reduced energy intake and increased expenditure leads to caloric deficits, which can be as extreme as 1,200 kcal/day (5, 6). Patients with cancer who present with weight loss also have reduced synthesis of muscle proteins; this highlights the importance of reduced dietary intake in the pathogenesis of cancer-associated sarcopenia and cachexia, and stresses the crucial role of generating an anabolic response by supplementation of nutrients able to reactivate protein synthesis (5, 6, 10, 11). Several clinical practice guidelines provide recommendations for the clinical management of cancer cachexia (12–14). Both the European Society for Clinical Nutrition and Metabolism (ESPEN) and European Society for Medical Oncology (ESMO) guidelines provide recommendations on energy and protein requirements, and estimate that total energy expenditure in patients with cancer falls in the range of 25–30 kcal/kg/day (12, 13). Protein intake recommendations set the minimum protein supply at 1 g/kg/day, with a target supply of 1.2–2 g/kg/day (12, 13, 15).

As cancer progresses, energy and protein intake are expected to deteriorate. However, the lack of awareness by many physicians regarding the nutritional status of patients frequently results in progressive and underestimated weight loss until it becomes severe and scarcely treatable (2, 16). In patients with certain types of cancer, such as gastroesophageal cancer, nutritional depletion has been detected already at early disease stages (17), underscoring the need for an early multimodal approach aimed at prevention, early recognition, and treatment of the metabolic and nutritional derangements (18). A reduction in food intake needs to be recognized early and promptly managed, and oral energy intake should be assessed at least qualitatively and, if possible, quantitatively (19).

The aims of the current study were (1) to evaluate the prevalence of anorexia and hypophagia in patients with gastrointestinal or lung cancers at the time of diagnosis, (2) to compare energy and protein intake of patients with guideline recommendations, (3) to assess the prevalence of pre-cachexia and cachexia, and (4) to determine whether nutritional impairments were already present in these patients prior to any therapeutic intervention. In addition, potential correlations between dietary intake and weight loss were explored.

## Patients and methods

### Patients

Eligible patients (>18 years) were newly diagnosed with gastrointestinal tract or lung tumors and naive to any oncologic treatment (e.g., chemo- or radiotherapy and surgery). Exclusion criteria included oral feeding incapacity, dysphagia, intestinal obstruction or occlusion, severe liver failure (total bilirubin >1.5 mg/dl, and aspartate aminotransferase/alanine aminotransferase >2 × upper limit of normal or in the case of metastatic liver >5 × upper limit of normal), severe kidney failure (creatinine >2.0 mg/dl and creatinine clearance <50 ml/min), acute decompensated heart failure, active infection, primary or metastatic brain tumor, severe psychiatric disorders, and Mini-Mental State Examination <25/30 in patients >70 years of age.

### Study design

This prospective, non-interventional study complied with the principles of the Declaration of Helsinki amended in 2013 and received ethics committee approval at all participating institutions. The study was approved by the local ethics committee at the Campus Bio-Medico University, Rome, Italy. Informed consent was obtained from all patients. During the predefined consecutive period of time from January 2016 to November 2017, all patients in a single center at their first oncology visit who met the eligibility criteria were enrolled.

### Assessments

#### Nutritional status

All patients were evaluated for height and weight, and body mass index (BMI) was calculated. Weight loss over the previous 6 months was recorded (as reported by the patients). The risk of malnutrition was evaluated in all patients using the Malnutrition Universal Screening Tool (MUST), and patients were classified as being at low (MUST score = 0), medium (MUST score = 1), or high (MUST score = 2) nutritional risk (20). Malnutrition was diagnosed on the basis of the Global Leadership Initiative on Malnutrition (GLIM) criteria (21), which requires at least one phenotypic and one etiologic criterion to be present. The phenotypic criteria include unintentional weight loss, low BMI, and low fat-free mass index; etiologic criteria include reduced food intake or assimilation, disease burden, and inflammatory condition. Herein, unintentional weight loss/low BMI were used as phenotypic criteria, and the presence of inflammation [C-reactive protein (CRP)]/reduced food intake or malabsorption as etiologic criteria to diagnose malnutrition.

#### Anorexia, pre-cachexia, cachexia, and dietary intake assessments

Anorexia was identified by a score of ≤30 on a modified version of the Anorexia/Cachexia Subscale of the Functional Assessment of Anorexia/Cachexia Therapy (FAACT) questionnaire (22, 23). Additionally, anorexia was assessed by a dedicated anorexia questionnaire (AQ) investigating the presence of early satiety, taste/smell alterations, meat aversion, and nausea/vomiting. Patients showing at least one symptom were considered positive for the presence of anorexia (24, 25).

Following standardized criteria, pre-cachexia was defined as ≤5% weight loss over the past 6 months, with anorexia (FAACT score ≤ 30) and inflammation (CRP > 10 mg/L) (26). Cachexia was defined according to Fearon et al. (7) as >5% weight loss over the past 6 months, or BMI <20 kg/m^2^ and >2% weight loss.

Trained dietitians collected dietary intake data using a 3 days food record conducted on two non-consecutive weekdays and a weekend day. Energy and protein requirements were estimated following ESPEN and ESMO guidelines: 25–30 kcal/kg of body weight per day for energy intake and 1.2 g/kg of body weight per day for protein intake (12, 13). Hypophagia was defined as an energy intake of ≤70% with respect to the 30 kcal/kg recommendation.

### Statistical analysis

A descriptive analysis was performed. Qualitative variables were presented as absolute frequencies and percentages. The normality of the distribution of the continuous quantitative variables was evaluated through the Shapiro-Wilk test; the variables with Gaussian distribution were reported as mean and standard deviation (SD), and those with non-Gaussian distribution as median and interquartile range.

The Mann-Whitney non-parametric test was used to compare daily energy and protein intake of patients with those recommended in ESPEN and ESMO guidelines (12, 13). Correlation analysis was performed using the Pearson correlation coefficient.

A *p*-value < 0.05 was considered statistically significant and all tests were two-sided. All statistical analyses were performed with the software open-source R version 3.5.1.

## Results

In total, 102 patients with cancer (53 male; median age 67 years, range 21–88) were enrolled. Demographic and clinical characteristics of patients at time of diagnosis are shown in Table 1. Most patients (>80%) had a gastrointestinal tumor, namely gastroesophageal, pancreatic/biliary tract, or colorectal. Overall, 66% (67/102) of patients presented with advanced cancer (stage III–IV), which was more prevalent among patients with gastroesophageal (76%; 19/25) and colorectal tumors (72%; 13/18).

**TABLE 1:** Demographic and clinical characteristics at diagnosis.

Characteristic	Overall population *N* = 102	Gastroesophageal cancer *n* = 25	Pancreatic/Biliary tract cancer *n* = 42	Colorectal cancer *n* = 18	Lung cancer *n* = 17
Proportion of total population, %	100	24.5	41.1	17.6	16.7
**Gender, *n* (%)**					
Male	53 (51.9)	12 (48.0)	18 (42.9)	10 (55.6)	13 (76.5)
Female	49 (48.0)	13 (52.0)	24 (57.1)	8 (44.4)	4 (23.5)
Age, median (range)	67 (21–88)	67 (36–88)	72 (40–83)	65.5 (31–82)	63.5 (21–83)
**Cancer stage, *n* (%)**					
I	10 (9.8)	0	3 (7.1)	2 (11.1)	5 (29.4)
II	25 (24.5)	6 (24.0)	15 (35.7)	3 (16.7)	1 (5.9)
III	18 (17.6)	6 (24.0)	5 (11.9)	4 (22.2)	3 (17.6)
IV	49 (48.0)	13 (52.0)	19 (45.2)	9 (50.0)	8 (47.1)
BMI, kg/m^2^, mean (SD)	23.1 (3.4)	22.5 (3.4)	23.0 (3.4)	23.9 (2.7)	23.4 (3.6)
BMI < 20 kg/m^2^, *n* (%)	16 (15.7)	5 (20.0)	8 (19.0)	0	3 (17.6)
WL, kg, mean (SD)	7.6 (6.0)	8.0 (5.6)	8.2 (5.8)	6.2 (5.3)	6.6 (7.0)
WL, %, mean (SD)	10.1 (7.8)	11.1 (7.3)	11.0 (7.5)	8.1 (7.1)	8.8 (9.0)
6 months WL > 5%, *n* (%)	70 (68.6)	17 (68.0)	30 (71.4)	11 (61.1)	12 (70.6)
CRP^a,b^, mg/L, mean (SD)	16.6 (18.4)	19.0 (22.2)	18.5 (17.5)	11.6 (10.9)	14.7 (19.6)
**Malnutrition risk (MUST), *n* (%)**					
Low	27 (26.5)	6 (24.0)	10 (23.8)	5 (27.7)	6 (35.3)
Medium	24 (23.5)	4 (16.0)	7 (16.6)	9 (50.0)	4 (23.5)
High	51 (50.0)	15 (60.0)	25 (59.5)	4 (22.2)	7 (41.2)
**Patients with, *n* (%)**					
Malnutrition (GLIM)	61 (59.8)	15 (60.0)	30 (71.4)	9 (50.0)	7 (41.2)
Pre-cachexia	11 (10.7)	2 (8.0)	2 (4.8)	3 (16.7)	4 (23.5)
Cachexia	69 (67.6)	18 (72.0)	31 (73.8)	10 (55.6)	10 (58.8)

^a^A CRP of 10 mg/L was considered the upper limit of normality.

^b^CRP levels were not determined for four patients (*n* = 2 for gastroesophageal cancer; *n* = 1 pancreatic/biliary tract cancer; *n* = 1 lung cancer). BMI, body mass index; CRP, C-reactive protein; GLIM, Global Leadership Initiative on Malnutrition; MUST, Malnutrition Universal Screening Tool; SD, standard deviation; WL, weight loss.

### Nutritional status

The mean BMI ± SD in the overall population was 23.1 ± 3.4 kg/m^2^ and, on average, patients had experienced weight loss ± SD of 7.6 ± 6.0 kg in the previous 6 months. According to MUST scores, 26.5% (27/102) of patients were at low risk for malnutrition, 23.5% (24/102) were at medium risk, and 50% (51/102) were at high risk. Patients’ risk of malnutrition by cancer type is summarized in Table 1. Malnutrition was diagnosed in 59.8% (61/102) of patients, per GLIM criteria. The prevalence of malnutrition was highest among patients with pancreatic/biliary tract cancer (71.4%; 30/42), and lowest in patients with lung cancer (41.2%; 7/17) (Table 1).

### Prevalence of pre-cachexia/cachexia, anorexia, hypophagia, and weight loss

**FIGURE 1:**
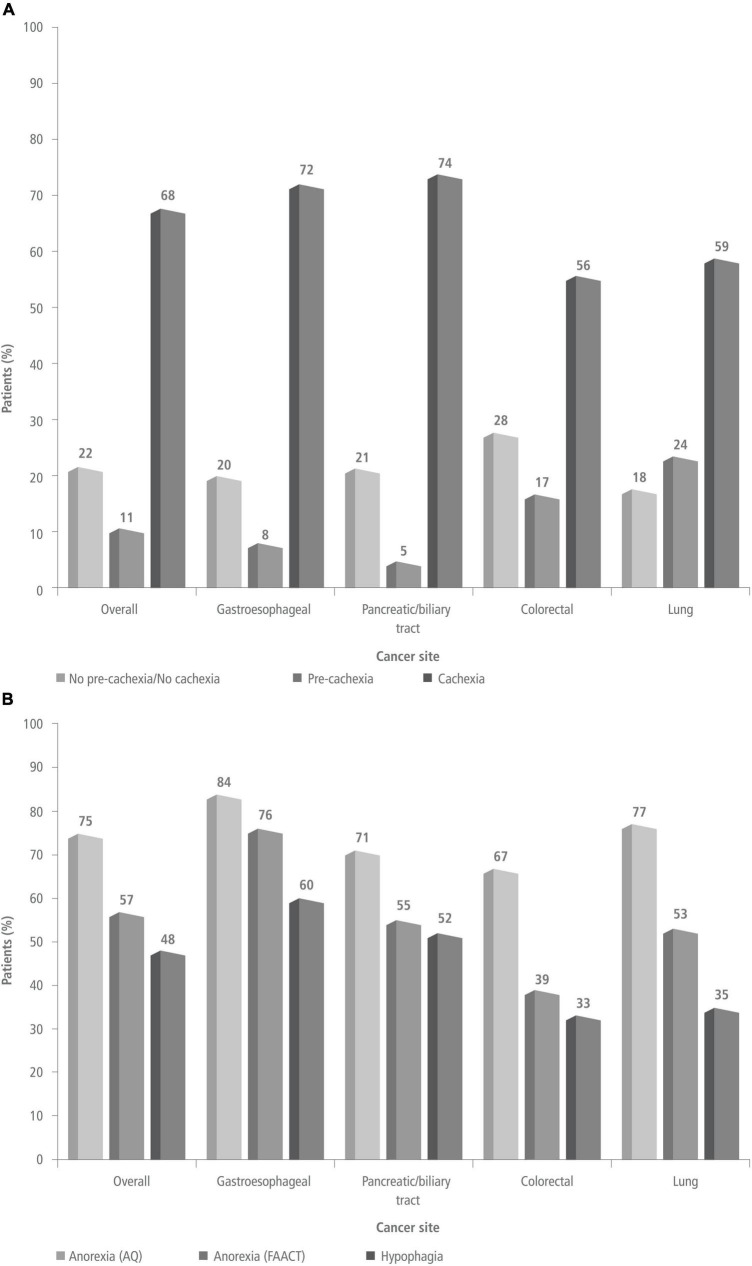
Prevalence of pre-cachexia/cachexia [panel **(A)**] and prevalence of anorexia and hypophagia [panel **(B)**] at the time of diagnosis in the overall study population and by cancer site. AQ, anorexia questionnaire; FAACT, functional assessment of anorexia/cachexia therapy.

At the time of diagnosis, 10.8% (11/102) of patients were pre-cachectic, 67.6% (69/102) were cachectic, and 21.6% (22/102) were not classifiable as pre-cachectic or cachectic (their weight was stable) (Figure 1A). Patients with pancreatic/biliary tract (73.8%; 31/42) or gastroesophageal cancers (72%; 18/25) had a higher prevalence of cachexia than patients with lung (58.8%; 10/17) or colorectal cancers (55.6%; 10/18) (Table 1). Anorexia was present in 56.8% (58/102) of patients per FAACT scores, and in 74.5% (76/102) per AQ results. Anorexia was most prevalent in patients with gastroesophageal cancer, and least prevalent in those with colorectal cancer (Figure 1B). Additionally, 48% (49/102) of patients had hypophagia, and involuntary weight loss in the prior 6 months was documented in 87 of 102 patients (85.2%). Among the patients with pre-cachexia, 7 (64%) were also hypophagic.

### Energy and protein intake

**FIGURE 2:**
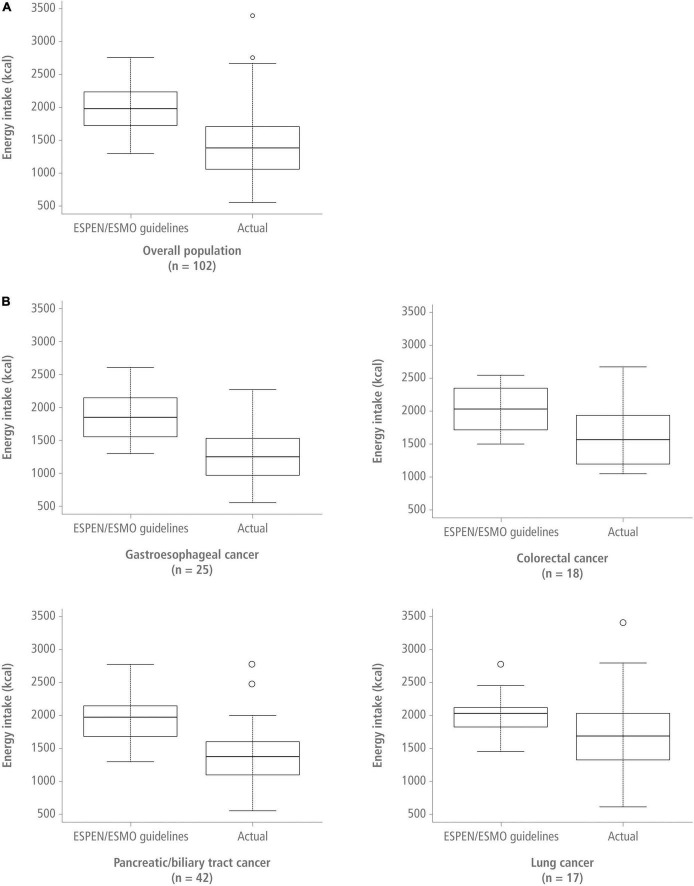
Recommended energy intake (upper limit) vs. actual energy intake in the overall population [panel **(A)**] and for the different cancer sites [panel **(B)**] presented as kcal/day. ESMO, European Society for Medical Oncology; ESPEN, European Society for Clinical Nutrition and Metabolism.

In the overall population, patients had significantly lower energy intake compared with the recommended range (25–30 kcal/kg/day) (*p* < 0.00001) (Table 2 and Figure 2). Patients with gastroesophageal cancer had the lowest median energy intake [18.4 kcal/kg/day (13.4–25.6)], whereas patients with colorectal cancer had the highest [23.7 kcal/kg (19.6–26.3)]. Energy intake was significantly below the recommended 30 kcal/kg/day in all patients, except for patients with lung cancer, whose energy intake was below the daily 30 kcal/kg recommendation but the difference did not reach statistical significance. Among patients with gastroesophageal and pancreatic/biliary tract tumors, energy intake was also significantly below the recommended 25 kcal/kg/day limit. Protein intake was significantly below the 1.2 g/kg target in all patient populations, with median protein intake levels being lowest in patients with gastroesophageal cancer (Table 3).

**TABLE 2:** Actual daily energy intake and European Society for Clinical Nutrition and Metabolism (ESPEN)/European Society for Medical Oncology (ESMO)-recommended energy intake.

Population	Median energy intake, kcal/kg (IQR)	Percentage of recommended 25 kcal/kg (IQR)	*P*-value (vs. 25 kcal/kg)	Percentage of recommended 30 kcal/kg (IQR)	*P*-value (vs. 30 kcal/kg)
Overall (*n* = 102)	21.1 (17.3–26.9)	84.4 (69.2–107.6)	0.001	70.3 (57.7–89.7)	<0.001
Gastroesophageal cancer (*n* = 25)	18.4 (13.4–25.6)	73.6 (53.6–102.4)	0.014	61.3 (44.7–85.3)	<0.001
Pancreatic/biliary tract cancer (*n* = 42)	20.7 (17.3–24.9)	82.8 (69.2–99.6)	0.004	69.0 (57.7–83.0)	<0.001
Colorectal cancer (*n* = 18)	23.7 (19.6–26.3)	94.8 (78.4–105.2)	0.538	79.0 (65.3–87.7)	0.013
Lung cancer (*n* = 17)	21.8 (18.2–30.9)	99.0 (72.8–140.4)	1.0	72.7 (60.7–103.0)	0.066

ESMO, European Society for Medical Oncology; ESPEN, European Society for Clinical Nutrition and Metabolism; IQR, interquartile range.

**TABLE 3:** Actual daily protein intake and European Society for Clinical Nutrition and Metabolism (ESPEN)/European Society for Medical Oncology (ESMO)-recommended protein intake.

Population	Actual protein intake g/kg (IQR)	Percentage of recommended 1.2 g/kg (IQR)	*P*-value (vs. 1.2 g/kg)
Overall (*n* = 102)	0.9 (0.7–1.1)	75.0 (58.3–91.7)	<0.001
Gastroesophageal cancer (*n* = 25)	0.8 (0.6–1.1)	66.7 (50.0–91.7)	0.002
Pancreatic/biliary tract cancer (*n* = 42)	0.9 (0.7–1.2)	75.0 (58.3–100)	<0.001
Colorectal cancer (*n* = 18)	1.0 (0.8–1.2)	83.3 (66.7–100)	0.017
Lung cancer (*n* = 17)	0.9 (0.8–1.0)	75.0 (66.7–83.3)	0.016

ESMO, European Society for Medical Oncology; ESPEN, European Society for Clinical Nutrition and Metabolism; IQR, interquartile range.

### Daily dietary intake and weight loss in patients with and without anorexia

Patients with anorexia had a significantly lower median daily energy intake [1327.5 kcal/day, interquartile range (IQR): 965.5–1263.3] compared with patients without anorexia (1480.2 kcal/day, IQR: 1263.3–1911.0) (*p* = 0.002). Median daily protein intake was also significantly lower among patients with anorexia (55.0 g/day, IQR: 43–72 vs. 62.9 g/day, IQR: 51.3–78.7; *p* = 0.0257). However, no significant differences were observed between patients with and without anorexia in terms of median daily calorie intake/body weight (20.4 kcal/kg, IQR: 13–25.8 vs. 21.5 kcal/kg, IQR: 18.2–27.8, respectively; *p* = 0.064), and median daily protein intake/body weight (0.85 g/kg, IQR: 0.64–1.10 vs. 0.90 g/kg, IQR: 0.77–1.16, respectively; *p* = 0.242). The median percentage of weight loss over the previous 6 months in patients with anorexia was significantly greater (12.4%, IQR: 7.3–17.2) than in patients without anorexia (5.1%, IQR: 0.0–9.8; *p* = 0.0005).

### Daily dietary intake and weight loss correlations in patients with and without hypophagia

Among patients with hypophagia (*n* = 49), there was a significant negative correlation between total daily calorie (r = –0.40, *p* = 0.01) or protein (r = –0.340, *p* = 0.018) intake and percentage of weight loss. In patients without hypophagia (*n* = 53), no correlation was observed between total daily calorie (r = –0.067, *p* = 0.647) or protein (r = –0.047, *p* = 0.751) intake and percentage of weight loss.

## Discussion

The present study demonstrates a high prevalence of anorexia and hypophagia in patients newly diagnosed with cancer and naive to treatment. These patients also present malnutrition and an increased risk for it. Our results align with those in the PreMiO observational study enrolling nearly 2,000 patients with cancer, which reported a 51.1% prevalence of malnutrition (27). Furthermore, we show a high prevalence of cachexia, which was present in almost 70% of patients. Additionally, we show good consistency between high risk for malnutrition, presence of malnutrition, and presence of cachexia, even before anticancer treatment start.

Our study shows that, already at the time of cancer diagnosis, patients’ consumption of calories and protein is significantly lower than ESPEN/ESMO recommended values (12, 13), which may have contributed to increased weight loss and malnutrition. Previous studies have demonstrated that malnourishment can negatively impact clinical outcomes in patients with cancer (3, 4). Poor preoperative nutritional status negatively affected postoperative outcomes and was associated with longer hospital stay, while malnutrition correlated with lower tolerance to chemotherapeutic treatment and reduced survival (17, 28, 29). Reduced dietary intake and anorexia have been associated with advanced cancer stage (27, 29), and are main drivers for weight loss (30, 31). Early identification and treatment of reduced food intake and anorexia is recommended in clinical guidelines to potentially prevent weight loss and improve clinical outcomes (12–14). Nevertheless, further research on weight loss and anorexia is still needed (32). Our results highlight the clinical relevance of anorexia and hypophagia in weight loss in patients with cancer. These data are in line with an international study (*N* = 438) showing a prevalence of anorexia as high as 65.4% detected by AQ, and an association between anorexia and low food intake and weight loss over time (33). Our study revealed a negative correlation between percentage of weight loss and daily calorie or protein intake in patients with hypophagia, supporting the ESPEN/ESMO recommendations to increase dietary intake (12, 13).

Inflammation plays a key role in cancer cachexia (3). Pro-inflammatory cytokines secreted by tumor cells can activate the immune system to induce a systemic inflammatory response, which, if sustained can lead to chronic inflammation. A current study suggests that failure of the immune response to control tumor growth leads to cachexia as a tolerance defense mechanism. At the tolerance stage, cachexia is characterized by the presence of anorexia, anemia, and loss of skeletal muscle and adipose tissue aimed at limiting the tissue damage induced by the tumor and chronic inflammation (34). In our study, the mean values of the acute response protein CRP, indicative of an inflammatory response, were well above the upper limit of normality (10 mg/L) in the overall population (17 mg/L), and particularly among patients with gastroesophageal and pancreatic or biliary tract (19 mg/L for both) cancers.

The main limitation of the present study is that energy intake was calculated using 3-day food diaries in which patients recorded their food and fluid intake. This is a short time frame to capture food intake relative to the weight loss period and it does not identify day-to-day variations in diet. Nevertheless, longer food diaries were not feasible, given the goal to determine dietary intake unaffected by treatment. The use of appetite as a surrogate of food intake is not favored, as these two parameters are only moderately correlated, likely because appetite and food intake represent different aspects of food intake behavior. In fact, appetite is a dimension of ingestive behavior that also includes hunger and satiety, which together influence the food intake outcome (35).

Currently, there is no consensus on how to measure or define reduced food intake, and it has been classified as patient-reported reductions in food intake, or as energy intake below a measured energy expenditure or below the guideline-recommended energy and protein intakes (19, 36). Recently, web-based dietary tools have been developed with validity comparable to traditional methods and may reduce the burden for patients (37). However, despite the limitations of short-time food diaries, this study adds to a growing body of evidence that underscores the importance of identifying patients at risk for malnutrition early in the disease course and implementing appropriate intervention. Further research is warranted in larger patient populations and in a wider range of tumor types. The majority of patients in the current study had gastrointestinal cancers (*n* = 85), and only a limited number of patients with lung cancer (*n* = 17) were included. As such, the results for patients with lung cancer need to be interpreted with caution.

In conclusion, anorexia and inadequate nutritional intake are common in patients with gastrointestinal and lung cancer at time of diagnosis, suggesting that nutritional abnormalities may already be present at the onset of cancer. To prevent the detrimental effects of cachexia, healthcare providers should assess all patients for nutritional status at the earliest opportunity and on an ongoing basis, implementing nutritional interventions as part of routine care. Moreover, since a negative nutritional balance is progressively being recognized as a relevant pathogenic factor in cancer-related malnutrition and cachexia (3, 30, 38), multimodal strategies aimed at improving anorexia and food intake are urgently needed. The present study underscores the need for these interventions to be implemented in the early phases of cancer development.

## Data availability statement

The original contributions presented in this study are included in the article/supplementary material, further inquiries can be directed to the corresponding author.

## Ethics statement

The studies involving human participants were reviewed and approved by the Local Ethics Committee at the Campus Bio-Medico University, Rome, Italy. The patients/participants provided their written informed consent to participate in this study.

## Author contributions

AM, SE, and MM contributed to the conception and design of the study. SE organized the database. DS, MG, and AL enrolled the patients. CN elaborated the food diaries. MC reviewed the manuscript. AM, AG, and GI performed the statistical analysis. AM and MM wrote the first draft of the manuscript. All authors contributed to the manuscript revision, read, and approved the submitted version.
